# Current Awareness on Comparative and Functional Genomics

**DOI:** 10.1002/cfg.230

**Published:** 2003-10


**Comparative and Functional Genomics**
*Comp Funct Genom* 2003; **4**: 560-567
Published online in Wiley InterScience (www.interscience.wiley.com). **DOI:** 10.1002/cfg.230

### Current awareness on comparative and functional genomics

### 1 Reviews & symposia

2003. Special issue: Chemical genomics of anticancer agents. *Curr Med Chem* **10:** (9)

Adams MWW, Dailey HA, Delucas LJ, Luo M, Prestegard JH, Rose JP, Wang BC. 2003. The Southeast Collaboratory for Structural Genomics: A high-throughput gene to structure factory. *Account Chem Res* **36:** (3) 191.

Alaiya AA, Roblick UJ, Franzen B, Bruch HP, Auer G. 2003. Protein expression profiling in human lung, breast, bladder, renal, colorectal and ovarian cancers (Review). *J Chromatogr B* **787:** (1) 207.

Archakov AI, Govorun VM, Dubanov AV, Ivanov YD, Veselovsky AV, Lewi P, Janssen P. 2003. Protein-protein interactions as a target for drugs in proteomics (Review). *Proteomics* **3:** (4) 380.

Banci L, Rosato A. 2003. Structural genomics of proteins involved in copper homeostasis. *Account Chem Res* **36:** (3) 215.

Bennetzen JL, Ma JX. 2003. The genetic colinearity of rice and other cereals on the basis of genomic sequence analysis. *Curr Opin Plant Biol* **6:** (2) 128.

Bertucci F, Viens P, Tagett R, Nguyen C, Houlgatte R, Birnbaum D. 2003. DNA arrays in clinical oncology: Promises and challenges (Review). *Lab Invest* **83:** (3) 305.

Bunney WE, Bunney BG, Vawter MP, Tomita H, Li J, Evans SJ, Choudary PV, Myers RM, Jones EG, Watson SJ, Akil H. 2003. Microarray technology: A review of new strategies to discover candidate vulnerability genes in psychiatric disorders. *Am J Psychiatry* **160:** (4) 657.

Claverie JM, Monchois V, Audic S, Poirot O, Abergel C. 2002. In search of new anti-bacterial target genes: A comparative/structural genomics approach. *Comb Chem High Throughput Scr* **5:** (7) 511.

Collins FS, Green ED, Guttmacher AE, Guyer MS. 2003. A vision for the future of genomics research. *Nature* **422:** (6934) 835.

Delseny M. 2003. Towards an accurate sequence of the rice genome. *Curr Opin Plant Biol* **6:** (2) 101.

Dishman R. 2002. From genomics to proteomics: One approach. *Am Lab* **34:** (24) 28.

Dores RM, Lecaude S, Bauer D, Danielson PB. 2002. Analyzing the evolution of the opioid/orphanin gene family. *Mass Spectrom Rev* **21:** (4) 220.

Evans WE. 2003. Pharmacogenomics: Marshalling the human genome to individuals drug therapy. *Gut* **52:** (S2) II10.

Frank R. 2002. High-density synthetic peptide microarrays: Emerging tools for functional genomics and proteomics (Review). *Comb Chem High Throughput Scr* **5:** (6) 429.

Geschwind DH. 2003. DNA microarrays: Translation of the genome from laboratory to clinic (Review). *Lancet Neurol* **2:** (5) 275.

Grant BD, Wilkinson HA. 2003. Functional genomic maps in *Caenorhabditis elegans*. *Curr Opin Cell Biol* **15:** (2) 206.

Hamdan M, Righetti PG. 2002. Modern strategies for protein quantification in proteome analysis: Advantages and limitations. *Mass Spectrom Rev* **21:** (4) 287.

Han B, Xue YB. 2003. Genome-wide intraspecific DNA-sequence variations in rice. *Curr Opin Plant Biol* **6:** (2) 134.

Hao BL, Qi J, Wang B. 2003. Prokaryotic phylogeny based on complete genomes without sequence alignment (Review). *Mod Phys Lett B* **17:** (3) 91.

Hecker M, Engelmann S, Cordwell SJ. 2003. Proteomics of *Staphylococcus aureus*: Current state and future challenges (Review). *J Chromatogr B* **787:** (1) 179.

Heinemann U, Bussow K, Mueller U, Umbach P. 2003. Facilities and methods for the high-throughput crystal structural analysis of human proteins. *Account Chem Res* **36:** (3) 157.

Hood L. 2003. Systems biology: Integrating technology, biology, and computation. *Mech Ageing Dev* **124:** (1) 9.

Huang RP. 2003. Protein arrays, an excellent tool in biomedical research. *Front Biosci* **8:** (May) D559.

Hubalek M, Hernychova L, Havlasova J, Kasalova I, Neubauerova V, Stulik J, Macela A, Lundqvist M, Larsson P. 2003. Towards proteome database of *Francisella tularensis* (Review). *J Chromatogr B* **787:** (1) 149.

Izawa T, Takahashi Y, Yano M. 2003. Comparative biology comes into bloom: Genomic and genetic comparison of flowering pathways in rice and *Arabidopsis*. *Curr Opin Plant Biol* **6:** (2) 113.

Kaji H, Isobe T. 2003. Protein database of *Caenorhabditis elegans* (Review). *J Chromatogr B* **787:** (1) 91.

Kamme F, Erlander MG. 2003. Global gene expression analysis of single cells. *Curr Opin Drug Discov Dev* **6:** (2) 231.

Kanehisa M, Bork P. 2003. Bioinformatics in the post-sequence era. *Nat Genet* **33:** (Suppl) 305.

Kvasnicka F. 2003. Proteomics: General strategies and application to nutritionally relevant proteins (Review). *J Chromatogr B* **787:** (1) 77.

Kyogoku Y, Fujiyoshi Y, Shimada I, Nakamura H, Tsukihara T, Akutsu H, Odahara T, Okada T, Nomura N. 2003. Structural genomics of membrane proteins. *Account Chem Res* **36:** (3) 199.

Lefkovits I. 2003. Functional and structural proteomics: A critical appraisal (Review). *J Chromatogr B* **787:** (1) 1.

Liston A. 2002. Genomics, bioinformatics, and plant systematics. *Isr J Plant Sci* **50:** (Suppl) S89.

Lopez MF, Pluskal MG. 2003. Protein micro- and macroarrays: Digitizing the proteome (Review). *J Chromatogr B* **787:** (1) 19.

Madi A, Pusztahelyi T, Punyiczki M, Fesus L. 2003. The biology of the post-genomic era: The proteomics. *Acta Biol Hung* **54:** (1) 1.

Manabe T. 2003. Analysis of complex protein-polypeptide systems for proteomic studies (Review). *J Chromatogr B* **787:** (1) 29.

Musgrave D, Zhang XY, Dinger M. 2002. Archaeal genome organization and stress responses: Implications for the origin and evolution of cellular life. *Astrobiology* **2:** (3) 241.

Ong SE, Foster LJ, Mann M. 2003. Mass spectrometric-based approaches in quantitative proteomics. *Methods* **29:** (2) 124.

Paradkar A, Trefzer A, Chakraburtty R, Stassi D. 2003. *Streptomyces* genetics: A genomic perspective. *Crit Rev Biotechnol* **23:** (1) 1.

Patterson SD, Aebersold RH. 2003. Proteomics: The first decade and beyond. *Nat Genet* **33:** (Suppl) 311.

Pineiro C, Barros-Velazquez J, Vazquez J, Figueras A, Gallardo JM. 2003. Proteomics as a tool for the investigation of seafood and other marine products (Review). *J Proteome Res* **2:** (2) 127.

Pitarch A, Sanchez M, Nombela C, Gil C. 2003. Analysis of the *Candida albicans* proteome: I. Strategies and applications (Review). *J Chromatogr B* **787:** (1) 101.

Pitarch A, Sanchez M, Nombela C, Gil C. 2003. Analysis of the *Candida albicans* proteome: II. Protein information technology on the net (Update 2002) (Review). *J Chromatogr B* **787:** (1) 129.

Plowman JE. 2003. Proteomic database of wool components (Review). *J Chromatogr B* **787:** (1) 63.

Rosen R, Ron EZ. 2002. Proteome analysis in the study of the bacterial heat-shock response. *Mass Spectrom Rev* **21:** (4) 244.

Rupp B. 2003. High-throughput crystallography at an affordable cost: The TB Structural Genomics Consortium Crystallization Facility. *Account Chem Res* **36:** (3) 173.

In order to keep subscribers up-to-date with the latest developments in their field, this current awareness service is provided by John Wiley & Sons and contains newly-published material on comparative and functional genomics. Each bibliography is divided into 16 sections. 1 Reviews & symposia; 2 General; 3 Large-scale sequencing and mapping; 4 Evolutionary genomics; 5 Comparative genomics; 6 Pathways, gene families and regulons; 7 Pharmacogenomics; 8 EST, cDNA and other clone resources; 9 Functional genomics; 10 Transcriptomics; 11 Proteomics; 12 Protein structural genomics; 13 Metabolomics; 14 Genomic approaches to development; 15 Technological advances; 16 Bioinformatics. Within each section, articles are listed in alphabetical order with respect to author. If, in the preceding period, no publications are located relevant to any one of these headings, that section will be omitted.

Copyright © 2003 John Wiley & Sons, Ltd.

Satoh N. 2003. The ascidian tadpole larva: Comparative molecular development and genomics. *Nat Rev Genet* **4:** (4) 285.
Sawyer TK, Chorev M. 2003. Peptide revolution: Genomics, proteomics and therapeutics. *Biotechniques* **34:** (3) 594.
Schoof H, Karlowski WM. 2003. Comparison of rice and *Arabidopsis* annotation. *Curr Opin Plant Biol* **6:** (2) 106.
Schreiber S, Costello CM. 2002. Pharmacogenomics in gastrointestinal disorders. *Eur J Surg* **168:** (Suppl 587) 70.
Schwab W. 2003. Metabolome diversity: Too few genes, too many metabolites? (Review). *Phytochemistry* **62:** (6) 837.
Shaughnessy JD. 2003. Global gene expression profiling in the study of multiple myeloma. *Int J Hematol* **77:** (3) 213.
Staunton D, Owen J, Campbell ID. 2003. NMR and structural genomics. *Account Chem Res* **36:** (3) 207.
Sumner LW, Mendes P, Dixon RA. 2003. Plant metabolomics: Large-scale phytochemistry in the functional genomics era (Review). *Phytochemistry* **62:** (6) 817.
Toda T, Sugimoto M. 2003. Proteome analysis of Epstein-Barr virus-transformed B-lymphoblasts and the proteome database (Review). *J Chromatogr B* **787:** (1) 197.
Upreti RK, Kumar M, Shankar V. 2003. Bacterial glycoproteins: Functions, biosynthesis and applications (Review). *Proteomics* **3:** (4) 363.
Ureta-Vidal A, Ettwiller L, Birney E. 2003. Genome-wide analysis in metazoan eukaryotes. *Nat Rev Genet* **4:** (4) 251.
Vaughan DA, Morishima H, Kadowaki K. 2003. Diversity in the *Oryza* genus. *Curr Opin Plant Biol* **6:** (2) 139.
Vinga S, Almeida J. 2003. Alignment-free sequence comparison: A review. *Bioinformatics* **19:** (4) 513.
Vivanco F, Lopez-Bescos L, Tunon J, Egido J. 2003. Proteomics and cardiovascular disease (Review) (Spanish, English Abstract). *Rev Esp Cardiol* **56:** (3) 289.
Wakefield MJ, Graves JAM. 2003. The kangaroo genome: Leaps and bounds in comparative genomics (Review). *EMBO Rep* **4:** (2) 143.
Wang H, Hanash S. 2003. Multi-dimensional liquid phase based separations in proteomics (Review). *J Chromatogr B* **787:** (1) 11.
Ware D, Stein L. 2003. Comparison of genes among cereals. *Curr Opin Plant Biol* **6:** (2) 121.
Wilson DS, Nock S. 2003. Recent developments in protein microarray technology (Review). *Angew Chem Int Ed* **42:** (5) 494.
Wood DW. 2003. Simplified protein purification using engineered self-cleaving affinity tags. *J Chem Technol Biotechnol* **78:** (2-3) 103.
Wrigley CW, Batey, Il, Skylas DJ, Sharp PJ. 2003. Grain quality assessment using proteomics and genomics. *Food Aust* **55:** (4) 143.
Wulfkuhle JD, Liotta LA, Petricoin EF. 2003. Proteomic applications for the early detection of cancer. *Nat Rev Cancer* **3:** (4) 267.
Yee A, Pardee K, Christendat D, Savchenko A, Edwards AM, Arrowsmith CH. 2003. Structural proteomics: Toward high-throughput structural biology as a tool in functional genomics. *Account Chem Res* **36:** (3) 183.
Yelick PC, Schilling TF. 2002. Molecular dissection of craniofacial development using zebrafish. *Crit Rev Oral Biol Med* **13:** (4) 308.

### 3 Large-scale sequencing and mapping

Chowdhary BP, Raudsepp T, Kata SR, Goh G, Millon LV, Allan V, Piumi F, Guerin G, Swinburne J, Binns M *et al*. 2003. The first-generation whole-genome radiation hybrid map in the horse identifies conserved segments in human and mouse genomes. *Genome Res* **13:** (4) 742.
Cohen GN, Barbe V, Flament D, Galperin M, Heilig R, Lecompte O, Poch O, Prieur D, Querellou J, Ripp R *et al*. 2003. An integrated analysis of the genome of the hyperthermophilic archaeon *Pyrococcus abyssi*. *Mol Microbiol* **47:** (6) 1495.
Dumke R, Catrein I, Pirkl E, Herrmann R, Jacobs E. 2003. Subtyping of *Mycoplasma pneumoniae* isolates based on extended genome sequencing and on expression profiles. *Int J Med Microbiol* **292:** (7-8) 513.
Galagan JE, Calvo SE, Borkovich KA, Selker EU, Read ND, Jaffe D, FitzHugh W, Ma LJ, Smirnov S, Purcell S *et al*. 2003. The genome sequence of the filamentous fungus *Neurospora crassa*. *Nature* **422:** (6934) 859.
Hayashizaki Y. 2003. RIKEN mouse genome encyclopedia. *Mech Ageing Dev* **124:** (1) 93.
Li L, Brunk BP, Kissinger JC, Pape D, Tang KL, Cole RH, Martin J, Wylie T, Dante M, Fogarty SJ *et al*. 2003. Gene discovery in the Apicomplexa as revealed by EST sequencing and assembly of a comparative gene database. *Genome Res* **13:** (3) 443.
Mannhaupt G, Montrone C, Haase D, Mewes HW, Aign V, Hoheisel JD, Fartmann B, Nyakatura G, Kempken F, Maier J, Schulte U. 2003. What's in the genome of a filamentous fungus? Analysis of the *Neurospora* genome sequence. *Nucleic Acids Res* **31:** (7) 1944.
Ren SX, Gang F, Jiang XG, Zeng R, Miao YG, Xu H, Zhang YX, Xiong H, Lu G, Lu LF *et al*. 2003. Unique physiological and pathogenic features of *Leptospira interrogans* revealed by whole-genome sequencing. *Nature* **422:** (6934) 888.
Yuan YN, San Miguel PJ, Bennetzen JL. 2003. High-Cot sequence analysis of the maize genome. *Plant J* **34:** (2) 249.

### 4 Evolutionary genomics

Barrier M, Bustamante CD, Yu JY, Purugganan MD. 2003. Selection on rapidly evolving proteins in the *Arabidopsis* genome. *Genetics* **163:** (2) 723.
Ermolaeva MD, Wu M, Eisen JA, Salzberg SL. 2003. The age of the *Arabidopsis thaliana* genome duplication. *Plant Mol Biol* **51:** (6) 859.
Massey SE, Moura G, Beltrao P, Almeida R, Garey JR, Tuite MF, Santos MAS. 2003. Comparative evolutionary genomics unveils the molecular mechanism of reassignment of the CTG codon in *Candida* spp. *Genome Res* **13:** (4) 544.
Read TD, Myers GSA, Brunham RC, Nelson WC, Paulsen IT, Heidelberg J, Holtzapple E, Khouri H, Federova NB, Carty HA *et al*. 2003. Genome sequence of *Chlamydophila caviae* (*Chlamydia psittaci* GPIC): Examining the role of niche-specific genes in the evolution of the Chlamydiaceae. *Nucleic Acids Res* **31:** (8) 2134.

### 5 Comparative genomics

Alter O, Brown PO, Botstein D. 2003. Generalized singular value decomposition for comparative analysis of genome-scale expression data sets of two different organisms. *Proc Natl Acad Sci U S A* **100:** (6) 3351.
Bennett MD, Leitch IJ, Price HJ, Johnston JS. 2003. Comparison with *Caenorhabditis* (approx. 100Mb) and *Drosophila* (approx. 175 Mb) using flow cytometry show genome size in *Arabidopsis* to be approx. 157 Mb and thus approx. 25% larger than the *Arabidopsis* genome initiative estimate of ~125 Mb. *Ann Bot* **91:** (5) 547.
Boneca IG, De Reuse H, Epinat JC, Pupin M, Labigne A, Moszer I. 2003. A revised annotation and comparative analysis of *Helicobacter pylori* genomes. *Nucleic Acids Res* **31:** (6) 1704.
Buchrieser C, Rusniok C, Kunst F, Cossart P, Glaser P. 2003. Comparison of the genome sequences of *Listeria monocytogenes* and *Listeria innocua*: Clues for evolution and pathogenicity. *FEMS Immunol Med Microbiol* **35:** (3) 207.
Chang JS, Van Remmen H, Cornell J, Richardson A, Ward WF. 2003. Comparative proteomics: Characterization of a two-dimensional gel electrophoresis system to study the effect of aging on mitochondrial proteins. *Mech Ageing Dev* **124:** (1) 33.
Deng W, Liou SR, Plunkett G, Mayhew GF, Rose DJ, Burland V, Kodoyianni V, Schwartz DC, Blattner FR. 2003. Comparative genomics of *Salmonella enterica* serovar Typhi strains Ty2 and CT18. *J Bacteriol* **185:** (7) 2330.
Emes RD, Goodstadt L, Winter EE, Ponting CP. 2003. Comparison of the genomes of human and mouse lays the foundation of genome zoology. *Hum Mol Genet* **12:** (7) 701.
Georgi LL, Wang Y, Reighard GL, Mao L, Wing RA, Abbott AG. 2003. Comparison of peach and *Arabidopsis* genomic sequences: Fragmentary conservation of gene neighborhoods. *Genome* **46:** (2) 268.
Govorun VM, Moshkovskii SA, Tikhonova OV, Goufman EI, Serebryakova MV, Momynaliev KT, Lokhov PG, Khryapova EV, Kudryavtseva LV, Smirnova OV *et al*. 2003. Comparative analysis of proteome maps of *Helicobacter pylori* clinical isolates. *Biochemistry (Mosc)* **68:** (1) 42.
Liu GR, Edwards K, Eisenstark A, Fu YM, Liu WQ, Sanderson KE, Johnston RN, Liu SL. 2003. Genomic diversification among archival strains of *Salmonella enterica* serovar Typhimurium LT7. *J Bacteriol* **185:** (7) 2131.
Locke DP, Segraves R, Carbone L, Archidiacono N, Albertson DG, Pinkel D, Eichler EE. 2003. Large-scale variation among human and

Copyright © 2003 John Wiley & Sons, Ltd. 

great ape genomes determined by array comparative genomic hybridization. *Genome Res* **13:** (3) 347.
Marin I. 2003. Evolution of chromatin-remodeling complexes: Comparative genomics reveals the ancient origin of "novel" compensasome genes. *J Mol Evol* **56:** (5) 527.
Nandi T, Dash D, Ghai R, Rao CB, Kannan K, Brahmachari SK, Ramakrishnan C, Ramachandran S. 2003. A novel complexity measure for comparative analysis of protein sequences from complete genomes. *J Biomol Struct Dyn* **20:** (5) 657.
Simpson PJ, Stanton C, Fitzgerald GF, Ross RP. 2003. Genomic diversity and relatedness of bifidobacteria isolated from a porcine cecum. *J Bacteriol* **185:** (8) 2571.

### 6 Pathways, gene families and regulons

Begley TJ, Rosenbach AS, Ideker T, Samson LD. 2002. Damage recovery pathways in *Saccharomyces cerevisiae* revealed by genomic phenotyping and interactome mapping. *Mol Cancer Res* **1:** (2) 103.
Lifanov AP, Makeev VJ, Nazina AG, Papatsenko DA. 2003. Homotypic regulatory clusters in *Drosophila*. *Genome Res* **13:** (4) 579.
Peregrin-Alvarez JM, Tsoka S, Ouzounis CA. 2003. The phylogenetic extent of metabolic enzymes and pathways. *Genome Res* **13:** (3) 422.
Regelmann J, Schule T, Josupeit FS, Horak J, Rose M, Entian KD, Thumm M, Wolf DH. 2003. Catabolite degradation of fructose-1,6-bisphosphatase in the yeast *Saccharomyces cerevisiae*: A genome-wide screen identifies eight novel GID genes and indicates the existence of two degradation pathways. *Mol Biol Cell* **14:** (4) 1652.
Riedel K, Arevalo-Ferro C, Reil G, Gorg A, Lottspeich F, Eberl L. 2003. Analysis of the quorum-sensing regulon of the opportunistic pathogen *Burkholderia cepacia* H111 by proteomics. *Electrophoresis* **24:** (4) 740.
Sun JB, Van den Heuvel J, Soucaille P, Qu YB, Zeng AP. 2003. Comparative genomic analysis of *dha* regulon and related genes for anaerobic glycerol metabolism in bacteria. *Biotechnol Progr* **19:** (2) 263.
Tocchetti A, Confalonieri S, Scita G, Di Fiore PP, Betsholtz C. 2003. *In silico* analysis of the *EPS8* gene family: Genomic organization, expression profile, and protein structure. *Genomics* **81:** (2) 234.
Wagner VE, Bushnell D, Passador L, Brooks AI, Iglewski BH. 2003. Microarray analysis of *Pseudomonas aeruginosa* quorum-sensing regulons: Effects of growth phase and environment. *J Bacteriol* **185:** (7) 2080.
Zewail A, Xie MW, Xing Y, Lin L, Zhang PF, Zou W, Saxe JP, Huang J. 2003. Novel functions of the phosphatidylinositol metabolic pathway discovered by a chemical genomics screen with wortmannin. *Proc Natl Acad Sci U S A* **100:** (6) 3345.

### 7 Pharmacogenomics

Ahmed N, Oliva K, Wang Y, Quinn M, Rice G. 2003. Proteomic profiling of proteins associated with urokinase plasminogen activator receptor in a colon cancer cell line using an antisense approach. *Proteomics* **3:** (3) 288.
Ballot E, Bruneel A, Labas V, Johanet C. 2003. Identification of rat targets of anti-soluble liver antigen autoantibodies by serologic proteome analysis. *Clin Chem* **49:** (4) 634.
Blaxall BC, Tschannen-Moran BM, Milano CA, Koch WJ. 2003. Differential gene expression and genomic patient stratification following left ventricular assist device support. *J Am Coll Cardiol* **41:** (7) 1096.
Bruneau JM, Maillet I, Tagat E, Legrand R, Supatto F, Fudali C, LeCaer JP, Labas V, Lecaque D, Hodgson J. 2003. Drug induced proteome changes in *Candida albicans*: Comparison of the effect of β(1,3) glucan synthase inhibitors and two triazoles, fluconazole and itraconazole. *Proteomics* **3:** (3) 325.
Chen G, Gharib TG, Thomas DG, Huang CC, Misek DE, Kuick RD, Giordano TJ, Iannettoni MD, Orringer MB, Hanash SM, Beer DG. 2003. Proteomic analysis of eIF-5A in lung adenocarcinomas. *Proteomics* **3:** (4) 496.
De Vos S, Hofmann WK, Grogan TM, Krug U, Schrage M, Miller TP, Braun JG, Wachsman W, Koeffler HP, Said JW. 2003. Gene expression profile of serial samples of transformed B-cell lymphomas. *Lab Invest* **83:** (2) 271.
Di Giovanni S, Knoblach SM, Brandoli C, Aden SA, Hoffman EP, Faden AI. 2003. Gene profiling in spinal cord injury shows role of cell cycle neuronal death. *Ann Neurol* **53:** (4) 454.
Dumur CI, Dechsukhum C, Ware JL, Cofield SS, Best AM, Wilkinson DS, Garrett CT, Ferreira-Gonzalez A. 2003. Genome-wide detection of LOH in prostate cancer using human SNP microarray technology. *Genomics* **81:** (3) 260.
Edvardsson U, Von Lowenhielm HB, Panfilov O, Nystrom AC, Nilsson F, Dahllof B. 2003. Hepatic protein expression of lean mice and obese diabetic mice treated with peroxisome proliferator-activated receptor activators. *Proteomics* **3:** (4) 468.
Guda K, Cui HY, Garg S, Dong M, Nambiar PR, Achenie LEK, Rosenberg DW. 2003. Multistage gene expression profiling in a differentially susceptible mouse colon cancer model. *Cancer Lett* **191:** (1) 17.
Gururaja TL, Li W, Payan DG, Anderson DC. 2002. Utility of peptide-protein affinity complexes in proteomics: Identification of interaction partners of a tumor suppressor peptide. *J Pept Res* **61:** (4) 163.
Heijne WHM, Stierum RH, Slijper M, Van Bladeren PJ, Van Ommen B. 2003. Toxicogenomics of bromobenzene hepatotoxicity: A combined transcriptomics and proteomics approach. *Biochem Pharmacol* **65:** (5) 857.
Hikkel I, Lucau-Danila A, Delaveau T, Marc P, Devaux F, Jacq C. 2003. A general strategy to uncover transcription factor properties identifies a new regulator of drug resistance in yeast. *J Biol Chem* **278:** (13) 11427.
Hwang D, Alevizos I, Schmitt WA, Misra J, Ohyama H, Todd R, Mahadevappa M, Warrington JA, Stephanopoulos G, Wong DT, Stephanopoulos G. 2003. Genomic dissection for characterization of cancerous oral epithelium tissues using transcription profiling. *Oral Oncol* **39:** (3) 259.
Iacobuzio-Donahue CA, Maitra A, Olsen M, Lowe AW, Van Heek NT, Rosty C, Walter K, Sato N, Parker A, Ashfaq R *et al*. 2003. Exploration of global gene expression patterns in pancreatic adenocarcinoma using cDNA microarrays. *Am J Pathol* **162:** (4) 1151.
Ito T, Okuma K, Ma XX, Yuzawa H, Hiramatsu K. 2003. Insights on antibiotic resistance of *Staphylococcus aureus* from its whole genome: Genomic island SCC. *Drug Resist Update* **6:** (1) 41.
Jiang YD, Zhang W, Kondo K, Klco JM, St Martin TB, Dufault MR, Madden SL, Kaelin WG, Nacht M. 2003. Gene expression profiling in a renal cell carcinoma cell line: Dissecting VHL and hypoxia-dependent pathways. *Mol Cancer Res* **1:** (6) 453.
Kikuchi T, Daigo Y, Katagiri T, Tsunoda T, Okada K, Kakiuchi S, Zembutsu H, Furukawa Y, Kawamura M, Kobayashi K, Imai K, Nakamura Y. 2003. Expression profiles of non-small cell lung cancers on cDNA microarrays: Identification of genes for prediction of lymph-node metastasis and sensitivity to anti-cancer drugs. *Oncogene* **22:** (14) 2192.
Kniazev YP, Cheburkin YV, Spikermann K, Peter S, Jenster G, Bangma KH, Karelin MI, Shkol'nik MI, Urbanskii AI, Evtushenko VI, Ullrich A, Kniazev PG. 2003. The cDNA microarray profiling of protein kinase and phosphatases: Molecular portrait of human prostate carcinomas. *Mol Biol-Engl Tr* **37:** (1) 89.
Kumar Y, Tatu U. 2003. Stress, protein flux during recovery from simulated ischemia: Induced heat shock protein 70 confers cytoprotection by suppressing JNK activation and inhibiting apoptotic cell death. *Proteomics* **3:** (4) 513.
Lame MW, Jones AD, Wilson DW, Segall HJ. 2003. Protein targets of 1,4-benzoquinone and 1,4-naphthoquinone in human bronchial epithelial cells. *Proteomics* **3:** (4) 479.
Li N, Mak A, Richards DP, Naber C, Keller BO, Li L, Shaw ARE. 2003. Monocyte lipid rafts contain proteins implicated in vesicular trafficking and phagosome formation. *Proteomics* **3:** (4) 536.
Li YW, Reichert WM. 2003. Adapting cDNA microarray format to cytokine detection protein arrays. *Langmuir* **19:** (5) 1557.
Li YW, Li XL, Sarkar FH. 2003. Gene expression profiles of $^{13}$C-and DIM-treated PC3 human prostate cancer cells determined by cDNA microarray analysis. *J Nutr* **133:** (4) 1011.
Lichtenfels R, Kellner R, Atkins D, Bukur J, Ackermann A, Beck J, Brenner W, Melchior S, Seliger B. 2003. Identification of metabolic enzymes in renal cell carcinoma utilizing PROTEOMEX analyses. *Biochim Biophys Acta* **1646:** (1-2) 21.
Liu LX, Liu ZH, Jiang HC, Zhang WH, Qi SY, Hu J, Wang XQ, Wu M. 2003. Gene expression profiles of hepatoma cell line HLE. *World J Gastroenterol* **9:** (4) 683.
Man K, Lo CM, Lee TKW, Li XL, Ng IOL, Fan ST. 2003. Intragraft gene expression profiles by cDNA microarray in small-for-size liver

Copyright © 2003 John Wiley & Sons, Ltd. 

grafts. *Liver Transplant* **9:** (4) 425.
Matsui H, Suzuki K, Hasumi M, Koike H, Okugi H, Nakazato H, Yamanaka H. 2003. Gene expression profiles of human BHP (II): Optimization of laser-capture microdissection and utilization of cDNA microarray. *Anticancer Res* **23:** (1A) 195.
Migliaccio S, Marino M. 2003. Estrogens and estrogen receptors: New actors in the plot of transcriptional regulation of genomic responses. *Calcif Tissue Int* **72:** (3) 181.
Mischel PS, Shai R, Shi T, Horvath S, Lu KV, Choe G, Seligson D, Kremen TJ, Palotie A, Liau LM, Cloughesy TF, Nelson SF. 2003. Identification of molecular subtypes of glioblastoma by gene expression profiling. *Oncogene* **22:** (15) 2361.
Nakajima F, Nishimori H, Hata F, Yasoshima T, Nomura H, Tanaka H, Ohno K, Yanai Y, Kamiguchi K, Sato N, Denno R, Hirata K.. 2003. Gene expression screening using a cDNA macroarray to clarify the mechanisms of peritoneal dissemination of pancreatic cancer. *Surg Today* **33:** (3) 190.
Nicolls MR, D'Antonio JM, Hutton JC, Gill RG, Czwornog JL, Duncan MW. 2003. Proteomics as a tool for discovery: Proteins implicated in Alzheimer's disease are highly expressed in normal pancreatic islets. *J Proteome Res* **2:** (2) 199.
Nishiyama N, Koizumi F, Okazaki S, Matsumura Y, Nishio K, Kataoka K. 2003. Differential gene expression profile between PC-14 cells treated with free cisplatin and cisplatin-incorporated polymeric micelles. *Bioconjug Chem* **14:** (2) 449.
Philip-Couderc P, Smih F, Pelat M, Vidal C, Verwaerde P, Pathak A, Buys S, Galinier M, Senard JM, Rouet P. 2003. Cardiac transcriptome analysis in obesity-related hypertension. *Hypertension* **41:** (3) 414.
Rao DV, Boyle GM, Parsons PG, Watson K, Jones GL. 2003. Influence of ageing, heat shock treatment and *in vivo* total antioxidant status on gene-expression profile and protein synthesis in human peripheral lymphocytes. *Mech Ageing Dev* **124:** (1) 55.
Rhoades K, Wong-Staal F. 2003. Inverse genomics™ as a powerful tool to identify novel targets for the treatment of neurodegenerative diseases. *Mech Ageing Dev* **124:** (1) 125.
Rodriguez S, Jafer O, Goker H, Summersgill BM, Zafarana G, Gillis AJM, Van Gurp RJHLM, Oosterhuis JW, Lu YJ, Huddart R *et al.* 2003. Expression profile of genes from 12p in testicular germ cell tumors of adolescents and adults associated with i(12p) and amplification at 12p11.2-p12.1. *Oncogene* **22:** (12) 1880.
Rogers PD, Barker KS. 2003. Genome-wide expression profile analysis reveals coordinately regulated genes associated with stepwise acquisition of azole resistance in *Candida albicans* clinical isolates. *Antimicrob Agents Chemother* **47:** (4) 1220.
Sabounchi-Schutt F, Astrom J, Hellman U, Eklund A, Grunewald J. 2003. Changes in bronchoalveolar lavage fluid proteins in sarcoidosis: A proteomics approach. *Eur Resp J* **21:** (3) 414.
Santamaria E, Avila MA, Latasa MU, Rubio A, Martin-Duce A, Lu SC, Mato JM, Corrales FJ. 2003. Functional proteomics of nonalcoholic steatohepatitis: Mitochondrial proteins as targets of *S*-adenosylmethionine. *Proc Natl Acad Sci U S A* **100:** (6) 3065.
Savoie CJ, Aburatani S, Watanabe S, Eguchi Y, Muta S, Imoto S, Miyano S, Kuhara S, Tashiro K. 2003. Use of gene networks from full genome microarray libraries to identify functionally relevant drug-affected genes and gene regulation cascades. *DNA Res* **10:** (1) 19.
Scherer A, Krause A, Walker JR, Sutton SE, Seron D, Raulf F, Cooke MP. 2003. Optimized protocol for linear RNA amplification and application to gene expression profiling of human renal biopsies. *Biotechniques* **34:** (3) 546.
Schlingemann J, Hess J, Wrobel G, Breitenbach U, Gebhardt C, Steinlein P, Kramer H, Furstenberger G, Hahn M, Angel P, Lichter P. 2003. Profile of gene expression induced by the tumour promotor TPA in murine epithelial cells. *Int J Cancer* **104:** (6) 699.
Tan ZJ, Hu XG, Cao GS, Tang Y. 2003. Analysis of gene expression profile of pancreatic carcinoma using cDNA microarray. *World J Gastroenterol* **9:** (4) 818.
Tang Y, Nee AC, Lu AG, Ran RQ, Sharp FR. 2003. Blood genomic expression profile for neuronal injury. *J Cereb Blood Flow Metab* **23:** (3) 310.
Tao W, Hangoc G, Hawes JW, Si Y, Cooper S, Broxmeyer HE. 2003. Profiling of differentially expressed apoptosis-related genes by cDNA arrays in human cord blood $CD34^+$ cells treated with etoposide. *Exp Hematol* **31:** (3) 251.
Van Duin M, Woolson H, Mallinson D, Black D. 2003. Genomics in target and drug discovery. *Biochem Soc Trans* **31:** (2) 429.
Virolle T, Krones-Herzig A, Baron V, De Gregorio G, Adamson ED, Mercola D. 2003. Egr1 promotes growth and survival of prostate cancer cells: Identification of novel Egr1 target genes. *J Biol Chem* **278:** (14) 11802.
Wang GQ, Zhang Y, Chen B, Cheng J. 2003. Preliminary studies on Alzheimer's disease using cDNA microarrays. *Mech Ageing Dev* **124:** (1) 115.
Wang HT, Kong JP, Ding F, Wang XQ, Wang MR, Liu LX, Wu M, Liu ZH. 2003. Analysis of gene expression profile induced by EMP-1 in esophageal cancer cells using cDNA microarray. *World J Gastroenterol* **9:** (3) 392.
Weekes J, Watson RR, Dunn MJ. 2003. Murine retrovirus infection and the effect of chronic alcohol consumption: Proteomic analysis of cardiac protein expression. *Alcohol Alcohol* **38:** (2) 103.
Weiss MM, Kuipers EJ, Postma C, Snijders AM, Siccama I, Pinkel D, Westerga J, Meuwissen SGM, Albertson DG, Meijer GA. 2003. Genomic profiling of gastric cancer predicts lymph node status and survival. *Oncogene* **22:** (12) 1872.
White IR, Man WJ, Bryant D, Bugelski P, Camilleri P, Cutler P, Hayes W, Holbrook JD, Kramer K, Lord PG, Wood J. 2003. Protein expression changes in the Sprague Dawley rat liver proteome following administration of peroxisome proliferator activated receptor $\alpha$ and $\gamma$ ligands. *Proteomics* **3:** (4) 505.
Williams NS, Gaynor RB, Scoggin S, Verma U, Gokaslan T, Simmang C, Fleming J, Tavana D, Frenkel E, Becerra C. 2003. Identification and validation of genes involved in the pathogenesis of colorectal cancer using cDNA microarrays and RNA interference. *Clin Cancer Res* **9:** (3) 931.
Wong LY, Hafeman A, Boyd VL, Bodeau J, Lazaruk KD, Liew SN, Casey P, Belonogoff V, Bit S, Sumner C *et al*, Baumhueter S. 2003. Assessing gene expression variation in normal human tissues using GeneTag™, a novel, global, sensitive profiling method. *J Biotechnol* **101:** (3) 199.
Woods AS, Moyer SC, Wang HYJ, Wise RA. 2003. Interaction of chlorisondamine with the neuronal nicotinic acetylcholine receptor. *J Proteome Res* **2:** (2) 207.
Yang SH, Kim JS, Oh TJ, Kim MS, Lee SW, Woo SK, Cho HS, Choi YH, Kim YH, Rha SY, Chung HC, An SW. 2003. Genome-scale analysis of resveratrol-induced gene expression profile in human ovarian cancer cells using a cDNA microarray. *Int J Oncol* **22:** (4) 741.
Ye QH, Qin LX, Forgues M, He P, Kim JW, Peng AC, Simon R, Li Y, Robles AI, Chen YD *et al*. 2003. Predicting hepatitis B virus-positive metastatic hepatocellular carcinomas using gene expression profiling and supervised machine learning. *Nat Med* **9:** (4) 416.
You SA, Archacki SR, Angheloiu G, Moravec CS, Rao SQ, Kinter M, Topol EJ, Wang Q. 2003. Proteomic approach to coronary atherosclerosis shows ferritin light chain as a significant marker: Evidence consistent with iron hypothesis in atherosclerosis. *Physiol Genomics* **13:** (1) 25.
Zachlederova M, Jarolim P. 2003. Gene expression profiles of microvascular endothelial cells after stimuli implicated in the pathogenesis of vasoocclusion. *Blood Cells Mol Dis* **30:** (1) 70.
Zhang LY, Ying WT, Mao YS, He HZ, Liu Y, Wang HX, Liu F, Wang K, Zhang DC, Wang Y *et al.* 2003. Loss of clusterin both in serum and tissue correlates with the tumorigenesis of esophageal squamous cell carcinoma *via* proteomics approaches. *World J Gastroenterol* **9:** (4) 650.
Zhao R, Ji JG, Tong YP, Pu H, Ru BG. 2003. Use of serological proteomic methods to find biomarkers associated with breast cancer. *Proteomics* **3:** (4) 433.
Zhou XH, Krueger JG, Kao MCJ, Lee E, Du FH, Menter A, Wong WH, Bowcock AM. 2003. Novel mechanisms of T-cell and dendritic cell activation revealed by profiling of psoriasis on the 63,100-element oligonucleotide array. *Physiol Genomics* **13:** (1) 69.

### 8 EST, cDNA and other clone resources

Bertani GR, Johnson RK, Robic A, Pomp D. 2003. Mapping of porcine ESTs obtained from the anterior pituitary. *Anim Genet* **34:** (2) 132.
Fu GK, Stuve LL. 2003. Construction of cDNA libraries for highly efficient DNA sequencing from the 3′ end of expressed genes. *Biotechniques* **34:** (4) 758.
Knietsch A, Waschkowitz T, Bowien S, Henne A, Daniel R. 2003. Construction and screening of metagenomic libraries derived from enrich-

Copyright © 2003 John Wiley & Sons, Ltd. 

ment cultures: Generation of a gene bank for genes conferring alcohol oxidoreductase activity on *Escherichia coli*. *Appl Environ Microbiol* **69:** (3) 1408.
Lee MK, Ren CW, Yan B, Cox B, Zhang HB, Romanov MN, Sizemore FG, Suchyta SP, Peters E, Dodgson JB. 2003. Construction and characterization of three BAC libraries for analysis of the chicken genome. *Anim Genet* **34:** (2) 151.
Lo J, Lee SC, Xu M, Liu F, Ruan H, Eun A, He YW, Ma WP, Wang WF, Wen ZL, Peng JR. 2003. 15,000 unique zebrafish EST clusters and their future use in microarray for profiling gene expression patterns during embryogenesis. *Genome Res* **13:** (3) 455.
Ogihara Y, Mochida K, Nemoto Y, Murai K, Yamazaki Y, Shinl T, Kohara Y. 2003. Correlated clustering and virtual display of gene expression patterns in the wheat life cycle by large-scale statistical analyses of expressed sequence tags. *Plant J* **33:** (6) 1001.
Shabaan AM, Mohamed MM, Abdallah MS, Ibrahim HM, Karim AM. 2003. Analysis of *Schistosoma mansoni* genes using the expressed sequence tag approach. *Acta Biochim Pol* **50:** (1) 259.
Trail F, Xu JR, SanMiguel P, Halgren RG, Kistler HC. 2003. Analysis of expressed sequence tags from *Gibberella zeae* (anamorph *Fusarium graminearum*). *Fungal Genet Biol* **38:** (2) 187.
Unneberg P, Wennborg EA, Larsson M. 2003. Transcript identification by analysis of short sequence tags-influence of tag length, restriction site and transcript database. *Nucleic Acids Res* **31:** (8) 2217.
Wipf D, Benjdia M, Rikirsch E, Zimmermann S, Tegeder M, Frommer WB. 2003. An expression cDNA library for suppression cloning in yeast mutants, complementation of a yeast his4 mutant, and EST analysis from the symbiotic basidiomycete *Hebeloma cylindrosporum*. *Genome* **46:** (2) 177.

### 9 Functional genomics

Aburatani S, Tashiro K, Savoie CJ, Nishizawa M, Hayashi K, Ito Y, Muta S, Yamamoto K, Ogawa M, Enomoto A *et al*. 2003. Discovery of novel transcription control relationships with gene regulatory networks generated from multiple-disruption full genome expression libraries. *DNA Res* **10:** (1) 1.
Agaphonov MO, Deev AV, Kim SY, Sohn JH, Choi ES, Ter-Avanesyan MD. 2003. A novel approach to isolation and functional characterization of genomic DNA sequences from the methylotrophic yeast *Hansenula polymorpha*. *Mol Biol-Engl Tr* **37:** (1) 74.
Arava Y, Wang YL, Storey JD, Liu CL, Brown PO, Herschlag D. 2003. Genome-wide analysis of mRNA translation profiles in *Saccharomyces cerevisiae*. *Proc Natl Acad Sci U S A* **100:** (7) 3889.
Conlon EM, Liu XS, Lieb JD, Liu JS. 2003. Integrating regulatory motif discovery and genome-wide expression analysis. *Proc Natl Acad Sci U S A* **100:** (6) 3339.
Galiakparov N, Tanne E, Sela I, Gafny R. 2003. Functional analysis of the grapevine virus A genome. *Virology* **306:** (1) 42.
Galibert F, Andre C. 2002. The canine genome: An alternative model for mammalian gene function analysis (French, English Abstract). *Bull Acad Natl Med* **186:** (8) 1489.
Geoffroy MC, Floquet S, Metais A, Nassif X, Pelicic V. 2003. Large-scale analysis of the *Meningococcus* genome by gene disruption: Resistance to complement-mediated lysis. *Genome Res* **13:** (3) 391.
Hakanssan S, Behrens K, Marko-Varga G, Lindberg H, Pierrou S, Koopmann W. 2003. Identification of genes and proteins regulated by interleukin-5 in human eosinophils using microarrays and two-dimensional electrophoresis/mass spectrometry. *Chest* **123:** (3) S374.
Horn C, Offen N, Nystedt S, Hacker U, Wimmer EA. 2003. *piggyBac*-based insertional mutagenesis and enhancer detection as a tool for functional insect genomics. *Genetics* **163:** (2) 647.
Lange BM, Ghassemian M. 2003. Genome organization in *Arabidopsis thaliana*: A survey for genes involved in isoprenoid and chlorophyll metabolism. *Plant Mol Biol* **51:** (6) 925.
Morelle S, Carbonnelle E, Nassif X. 2003. The REP2 repeats of the genome of *Neisseria meningitidis* are associated with genes coordinately regulated during bacterial cell interaction. *J Bacteriol* **185:** (8) 2618.
Page N, Gerard-Vincent M, Menard P, Beaulieu M, Azuma M, Dijkgraaf GJP, Li HJ, Marcoux J, Nguyen T, Dowse T, Sdicu AM, Bussey H. 2003. A *Saccharomyces cerevisiae* genome-wide mutant screen for altered sensitivity to K1 killer toxin. *Genetics* **163:** (3) 875.

### I0 Transcriptomics

Bauersachs S, Blum H, Mallok S, Wenigerkind H, Rief S, Prelle K, Wolf E. 2003. Regulation of ipsilateral and contralateral bovine oviduct epithelial cell function in the postovulation period: A transcriptomics approach. *Biol Reprod* **68:** (4) 1170.
Chen TB, Farragher S, Bjourson AJ, Orr DF, Rao P, Shaw C. 2003. Granular gland transcriptomes in stimulated amphibian skin secretions. *Biochem J* **371:** (1) 125.
Chinnusamy V, Ohta M, Kanrar S, Lee BH, Hong XH, Agarwal M, Zhu JK. 2003. ICE1: A regulator of cold-induced transcriptome and freezing tolerance in *Arabidopsis*. *Gene Dev* **17:** (8) 1043.
Dieck HT, Doring F, Roth HP, Daniel H. 2003. Changes in rat hepatic gene expression in response to zinc deficiency as assessed by DNA arrays. *J Nutr* **133:** (4) 1004.
Dobrindt U, Agerer F, Michaelis K, Janka A, Buchrieser C, Samuelson M, Svanborg C, Gottschalk G, Karch H, Hacker J. 2003. Analysis of genome plasticity in pathogenic and commensal *Escherichia coli* isolates by use of DNA arrays. *J Bacteriol* **185:** (6) 1831.
Draghici S, Khatri P, Martins RP, Ostermeier GC, Krawetz SA. 2003. Global functional profiling of gene expression. *Genomics* **81:** (2) 98.
Gnatenko DV, Dunn JJ, McCorkle SR, Weissmann D, Perrotta PL, Bahou WF. 2003. Transcript profiling of human platelets using microarray and serial analysis of gene expression. *Blood* **101:** (6) 2285.
Gonzalez R, Tao H, Purvis JE, York SW, Shanmugam KT, Ingram LO. 2003. Gene array-based identification of changes that contribute to ethanol tolerance in ethanologenic *Escherichia coli*: Comparison of KO11 (parent) to LY01 (resistant mutant). *Biotechnol Progr* **19:** (2) 612.
Grate LR, Bhattacharyya C, Jordan MI, Mian IS. 2003. Integrated analysis of transcript profiling and protein sequence data. *Mech Ageing Dev* **124:** (1) 109.
Kim H, Snesrud EC, Haas B, Cheung F, Town CD, Quackenbush J. 2003. Gene expression analyses of *Arabidopsis* chromosome 2 using a genomic DNA amplicon microarray. *Genome Res* **13:** (3) 327.
Kluger Y, Basri R, Chang JT, Gerstein M. 2003. Spectral biclustering of microarray data: Coclustering genes and conditions. *Genome Res* **13:** (4) 703.
Lee JM, Calkins MJ, Chan KM, Kan TW, Johnson JA. 2003. Identification of the NF-E2-related factor-2-dependent genes conferring protection against oxidative stress in primary cortical astrocytes using oligonucleotide microarray analysis. *J Biol Chem* **278:** (14) 12029.
Liu YQ, Zhou JZ, Omelchenko MV, Beliaev AS, Venkateswaran A, Stair J, Wu LY, Thompson DK, Xu D, Rogozin IB *et al*. 2003. Transcriptome dynamics of *Deinococcus radiodurans* recovering from ionizing radiation. *Proc Natl Acad Sci U S A* **100:** (7) 4191.
Milohanic E, Glaser P, Coppee JY, Frangeul L, Vega Y, Vazquez-Boland JA, Kunst F, Cossart P, Buchrieser C. 2003. Transcriptome analysis of *Listeria monocytogenes* identifies three groups of genes differently regulated by PrfA. *Mol Microbiol* **47:** (6) 1613.
Molle V, Nakaura Y, Shivers RP, Yamaguchi H, Losick R, Fujita Y, Sonenshein AL. 2003. Additional targets of the *Bacillus subtilis* global regulator CodY identified by chromatin immunoprecipitation and genome-wide transcript analysis. *J Bacteriol* **185:** (6) 1911.
Nunes LR, Rosato YB, Muto NH, Yanai GM, Da Silva VS, Leite DB, Goncalves ER, De Souza AA, Coletta-Filho HD, Machado MA, Lopes SA, De Oliveira RC. 2003. Microarray analyses of *Xylella fastidiosa* provide evidence of coordinated transcription control of laterally transferred elements. *Genome Res* **13:** (4) 570.
O'Connell BC, Cheung AF, Simkevich CP, Tam W, Ren XJ, Mateyak MK, Sedivy JM. 2003. A large scale genetic analysis of c-Myc-regulated gene expression patterns. *J Biol Chem* **278:** (14) 12563.
Ojaimi C, Brooks C, Casjens S, Rosa P, Elias A, Barbour A, Jasinskas A, Benach J, Katona L, Radolf J *et al*. 2003. Profiling of temperature-induced changes in *Borrelia burgdorferi* gene expression by using whole genome arrays. *Infect Immun* **71:** (4) 1689.
Olivero M, Ruggiero T, Coltella N, Maffe A, Calogero R, Medico E, Di Renzo MF. 2003. Amplification of repeat-containing transcribed sequences (ARTS): A transcriptome fingerprinting strategy to detect functionally relevant microsatellite mutations in cancer - Online art no. e33. *Nucleic Acids Res* **31:** (7) e33.
Pepe MS, Longton G, Anderson GL, Schummer M. 2003. Selecting differentially expressed genes from microarray experiments. *Biometrics* **59:** (1) 133.

Copyright © 2003 John Wiley & Sons, Ltd. 

Peters JE, Thate TE, Craig NL. 2003. Definition of the *Escherichia coli* MC4100 genome by use of a DNA array. *J Bacteriol* **185:** (6) 2017.
Polen T, Rittmann D, Wendisch VF, Sahm H. 2003. DNA microarray analyses of the long-term adaptive response of *Escherichia coli* to acetate and propionate. *Appl Environ Microbiol* **69:** (3) 1759.
Porwollik S, Frye J, Florea LD, Blackmer F, McClelland M. 2003. A non-redundant microarray of genes for two related bacteria. *Nucleic Acids Res* **31:** (7) 1869.
Rogers PD, Thornton J, Barker KS, McDaniel DO, Sacks GS, Swiatlo E, McDaniel LS. 2003. Pneumolysin-dependent and -independent gene expression identified by cDNA microarray analysis of THP-1 human mononuclear cells stimulated by *Streptococcus pneumoniae*. *Infect Immun* **71:** (4) 2087.
Schuster M, Lostroh CP, Ogi T, Greenberg EP. 2003. Identification, timing, and signal specificity of *Pseudomonas aeruginosa* quorum-controlled genes: A transcriptome analysis. *J Bacteriol* **185:** (7) 2066.
Stanley NR, Britton RA, Grossman AD, Lazazzera BA. 2003. Identification of catabolite repression as a physiological regulator of biofilm formation by *Bacillus subtilis* by use of DNA microarrays. *J Bacteriol* **185:** (6) 1951.
Stintzi A. 2003. Gene expression profile of *Campylobacter jejuni* in response to growth temperature variation. *J Bacteriol* **185:** (6) 2009.
Vilain C, Libert F, Venet D, Costagliola S, Vassart G. 2003. Small amplified RNA-SAGE: An alternative approach to study transcriptome from limiting amount of mRNA - Online article no. e24. *Nucleic Acids Res* **31:** (6) e24.
Yamagishi J, Isobe R, Takebuchi T, Bando H. 2003. DNA microarrays of baculovirus genomes: Differential expression of viral genes in two susceptible insect cell lines. *Arch Virol* **148:** (3) 587.

### I1 Proteomics

Bandow JE, Becher D, Buttner K, Hochgrafe F, Freiberg C, Brotz H, Hecker M. 2003. The role of peptide deformylase in protein biosynthesis: A proteomic study. *Proteomics* **3:** (3) 299.
Butt YKC, Lum JHK, Lo SCL. 2003. Proteomic identification of plant proteins probed by mammalian nitric oxide synthase antibodies. *Planta* **216:** (5) 762.
Dwyer DS, Ardizzone TD. 2003. Mimicry of dimerization by synthetic peptides designed to target homologous regions of proteins. *Proteomics* **3:** (3) 317.
Fountoulakis M, Gasser R. 2003. Proteomic analysis of the cell envelope fraction of *Escherichia coli*. *Amino Acids* **24:** (1-2) 19.
Galeva N, Yakovlev D, Koen Y, Duzhak T, Alterman M. 2003. Direct identification of cytochrome P450 isozymes by matrix-assisted laser desorption/ionization time of flight-based proteomic approach. *Drug Metab Dispos* **31:** (4) 351.
Garry RF, Dash S. 2003. Proteomics computational analyses suggest that hepatitis C virus E1 and pestivirus E2 envelope glycoproteins are truncated class II fusion proteins. *Virology* **307:** (2) 255.
Goshe MB, Blonder J, Smith RD. 2003. Affinity labeling of highly hydrophobic integral membrane proteins for proteome-wide analysis. *J Proteome Res* **2:** (2) 153.
Guillot A, Gitton C, Anglade P, Mistou MY. 2003. Proteomic analysis of *Lactococcus lactis*, a lactic acid bacterium. *Proteomics* **3:** (3) 337.
Hynes Sthn, McGuire J, Falt T, Wadstrom T. 2003. The rapid detection of low molecular mass proteins differentially expressed under biological stress for four *Helicobacter* spp. using ProteinChip® technology. *Proteomics* **3:** (3) 273.
Islam N, Tsujimoto H, Hirano H. 2003. Wheat proteomics: Relationship between fine chromosome deletion and protein expression. *Proteomics* **3:** (3) 307.
Islam N, Tsujimoto H, Hirano H. 2003. Proteome analysis of diploid tetraploid and hexaploid wheat: Towards understanding genome interaction in protein expression. *Proteomics* **3:** (4) 549.
Kiernan UA, Tubbs KA, Nedelkov D, Niederkofler EE, McConnell E, Nelson RW. 2003. Comparative urine protein phenotyping using mass spectrometric immunoassay. *J Proteome Res* **2:** (2) 191.
Konig S, Schmidt O, Rose K, Thanos S, Besselmann M, Zeller M. 2003. Sodium dodecyl sulfate *versus* acid-labile surfactant gel electrophoresis: Comparative proteomic studies on rat retina and mouse brain. *Electrophoresis* **24:** (4) 751.
Konishi H, Komatsu S. 2003. A proteomics approach to investigating promotive effects of brassinolide on lamina inclination and root growth in rice seedlings. *Biol Pharm Bull* **26:** (4) 401.
Kovarova H, Hajduch M, Livingstone M, Dzubak P, Lefkovits I. 2003. Analysis of state-specific phosphorylation of proteins by two-dimensional gel electrophoresis approach. *J Chromatogr B* **787:** (1) 53.
Liu YH, Vellekamp G, Chen GD, Mirza UA, Wylie D, Twarowska B, Tang JT, Porter FW, Wang SH, Nagabhushan TL, Pramanik BN. 2003. Proteomic study of recombinant adenovirus 5 encoding human p53 by matrix-assisted laser desorption/ionization mass spectrometry in combination with database search. *Int J Mass Spectrom* **226:** (1) 55.
Lupi A, Messana I, Denotti G, Schinina ME, Gambarini G, Fadda MB, Vitali A, Cabras T, Piras V, Patamia M, Cordaro M, Giardina B, Castagnola M. 2003. Identification of the human salivary cystatin complex by the coupling of high-performance liquid chromatography and ion-trap mass spectrometry. *Proteomics* **3:** (4) 461.
Ong SE, Kratchmarova I, Mann M. 2003. Properties of $^{13}$C-substituted arginine in stable isotope labeling by amino acids in cell culture (SILAC). *J Proteome Res* **2:** (2) 173.
Shen SH, Jing YX, Kuang TY. 2003. Proteomics approach to identify wound-response related proteins from rice leaf sheath. *Proteomics* **3:** (4) 527.
Svensson M, Skold K, Svenningsson P, Andren PE. 2003. Peptidomics-based discovery of novel neuropeptides. *J Proteome Res* **2:** (2) 213.
Talamo F, D'Ambrosio C, Arena S, Del Vecchio P, Ledda L, Zehender G, Ferrara L, Scaloni A. 2003. Proteins from bovine tissues and biological fluids: Defining a reference electrophoresis map for liver, kidney, muscle, plasma and red blood cells. *Proteomics* **3:** (4) 440.
Umar A, Luider TM, Berrevoets CA, Grootegoed JA, Brinkmann AO. 2003. Proteomic analysis of androgen-regulated protein expression in a mouse fetal vas deferens cell line. *Endocrinology* **144:** (4) 1147.
Wubbolts R, Leckie RS, Veenhuizen PTM, Schwarzmann G, Mobius W, Hoernschemeyer J, Slot JW, Geuze HJ, Stoorvogel W. 2003. Proteomic and biochemical analyses of human B cell-derived exosomes: Potential implications for their function and multivesicular body formation. *J Biol Chem* **278:** (13) 10963.
Yao XD, Afonso C, Fenselau C. 2003. Dissection of proteolytic 18O labeling: Endoprotease-catalyzed $^{16}$O-to-$^{18}$O exchange of truncated peptide substrates. *J Proteome Res* **2:** (2) 147.

### I2 Protein structural genomics

Rehm T, Huber R, Holak TA. 2002. Application of NMR in structural proteomics: Screening for proteins amenable to structural analysis. *Structure* **10:** (12) 1613.
Vincentelli R, Bignon C, Gruez A, Canaan S, Sulzenbacher G, Tegoni M, Campanacci V, Cambillau C. 2003. Medium-scale structural genomics: Strategies for protein expression and crystallization. *Account Chem Res* **36:** (3) 165.

### I3 Metabolomics

Castrillo JI, Hayes A, Mohammed S, Gaskell SJ, Oliver SG. 2003. An optimized protocol for metabolome analysis in yeast using direct infusion electrospray mass spectrometry. *Phytochemistry* **62:** (6) 929.
Yamazaki M, Nakajima J, Yamanashi M, Sugiyama M, Makita Y, Springob K, Awazuhara M, Saito K. 2003. Metabolomics and differential gene expression in anthocyanin chemo-varietal forms of *Perilla frutescens*. *Phytochemistry* **62:** (6) 987.

### I4 Genomic approaches to development

Baugh LR, Hill AA, Slonim DK, Brown EL, Hunter CP. 2003. Composition and dynamics of the *Caenorhabditis elegans* early embryonic transcriptome. *Development* **130:** (5) 889.
Fleet JC, Wang LY, Vitek O, Craig BA, Edenberg HJ. 2003. Gene expression profiling of Caco-2 BBe cells suggests a role for specific signaling pathways during intestinal differentiation. *Physiol Genomics* **13:** (1) 57.
Kawakami T, Nagata T, Muraguchi A, Nishimura T. 2003. Proteomic approach to apoptotic thymus maturation. *J Chromatogr B* **787:** (1) 223.
Ma LG, Zhao HY, Deng XW. 2003. Analysis of the mutational effects

Copyright © 2003 John Wiley & Sons, Ltd. 

of the COP/DET/FUS loci on genome expression profiles reveals their overlapping yet not identical roles in regulating *Arabidopsis* seedling development. *Development* **130:** (5) 969.
Michaut L, Flister S, Neeb M, White KP, CeRTA U, Gehring WJ. 2003. Analysis of the eye developmental pathway in *Drosophila* using DNA microarrays. *Proc Natl Acad Sci U S A* **100:** (7) 4024.
Tchuraev RN, Galimzyanov AV. 2003. On parametric stability of gene networks controlling ontogenetic processes. *Mol Biol-Engl Tr* **37:** (1) 81.
Voss SR, Prudic KL, Oliver JC, Shaffer HB. 2003. Candidate gene analysis of metamorphic timing in ambystomatid salamanders. *Mol Ecol* **12:** (5) 1217.
Yoda H, Momoi A, Esguerra CV, Meyer D, Driever W, Kondoh H, Furutani-Seiki M. 2003. An expression pattern screen for genes involved in the induction of the posterior nervous system of zebrafish. *Differentiation* **71:** (2) 152.

### I5 Technological advances

Ahram M, Flaig MJ, Gillespie JW, Duray PH, Linehan WM, Ornstein DK, Niu SL, Zhao YM, Petricoin EF, Emmert-Buck MR. 2003. Evaluation of ethanol-fixed, paraffin-embedded tissues for proteomic applications. *Proteomics* **3:** (4) 413.
Albert TJ, Norton J, Ott M, Richmond T, Nuwaysir K, Nuwaysir EF, Stengele KP, Green RD. 2003. Light-directed 5′→3′ synthesis of complex oligonucleotide microarrays - Online art no. e35. *Nucleic Acids Res* **31:** (7) e35.
Amon P, Ivanov I. 2003. Genomic DNA labeling for hybridization with DNA arrays. *Biotechniques* **34:** (4) 700.
Anderson DC, Li WQ, Payan DG, Noble WS. 2003. A new algorithm for the evaluation of shotgun peptide sequencing in proteomics: Support vector machine classification of peptide MS/MS spectra and SEQUEST scores. *J Proteome Res* **2:** (2) 137.
Banks RE, Craven RA, Harnden PA, Selby PJ. 2003. Technical brief: Use of a sensitive EnVision™ plus-based detection system for Western blotting - Avoidance of streptavidin binding to endogenous biotin and biotin-containing proteins in kidney and other tissues. *Proteomics* **3:** (4) 558.
Berka J, Ruiz-Martinez MC, Hammond R, Minarik M, Foret F, Sosic Z, Kleparnik K, Karger BL. 2003. Application of high-resolution capillary array 2 electrophoresis with automated fraction collection for GeneCalling™ analysis of the yeast genomic DNA. *Electrophoresis* **24:** (4) 639.
Charles PT, Vora GJ, Andreadis JD, Fortney AJ, Meador CE, Dulcey CS, Stenger DA. 2003. Fabrication and surface characterization of DNA microarrays using amine- and thiol-terminated oligonucleotide probes. *Langmuir* **19:** (5) 1586.
Clarke L, Millar BC, Moore JE. 2003. Extraction of genomic DNA from *Pseudomonas aeruginosa*: A comparison of three methods. *Br J Biomed Sci* **60:** (1) 34.
Cutler P, Heald G, White IR, Ruan J. 2003. A novel approach to spot detection for two-dimensional gel electrophoresis images using pixel value collection. *Proteomics* **3:** (4) 392.
Ding CM, Cantor CR. 2003. A high-throughput gene expression analysis technique using competitive PCR and matrix-assisted laser desorption/ionization time-of-flight MS. *Proc Natl Acad Sci U S A* **100:** (6) 3059.
Gotoh K, Oishi M. 2003. Screening of gene-associated polymorphisms by use of in-gel competitive reassociation and EST (cDNA) array hybridization. *Genome Res* **13:** (3) 492.
Havlis J, Thomas H, Sebela M, Shevchenko A. 2003. Fast-response proteomics by accelerated in-gel digestion of proteins. *Anal Chem* **75:** (6) 1300.
Huang GWS, Hong MY, Liu YC. 2003. Incorporation of DNA chip technology to the simulation and validation of flux analysis in yeast diauxic growth. *Life Sci* **72:** (22) 2525.
Janini GM, Conrads TP, Veenstra TD, Issaq HJ. 2003. Development of a two-dimensional protein-peptide separation protocol for comprehensive proteome measurements. *J Chromatogr B* **787:** (1) 43.
Jones LJ, Haugland RP, Singer VL. 2003. Development and characterization of the NanoOrange® protein quantitation assay: A fluorescence-based assay or proteins in solution. *Biotechniques* **34:** (4) 850.
Khan AH, Ossadtchi A, Leahy RM, Smith DJ. 2003. Error-correcting microarray design. *Genomics* **81:** (2) 157.
Khodarev NN, Park J, Kataoka Y, Nodzenski E, Hellman S, Roizman B, Weichselbaum RR, Pelizzari CA. 2003. Receiver operating characteristic analysis: A general tool for DNA array data filtration and performance estimation. *Genomics* **81:** (2) 202.
Klapa MI, Aon JC, Stephanopoulos G. 2003. Ion-trap mass spectrometry used in combination with gas chromatography for high-resolution metabolic flux determination. *Biotechniques* **34:** (4) 832.
Kusnezow W, Jacob A, Walijew A, Diehl F, Hoheisel JD. 2003. Antibody microarrays: An evaluation of production parameters. *Proteomics* **3:** (3) 254.
Levit-Binnun N, Lindner AB, Zik O, Eshhar Z, Moses E. 2003. Quantitative detection of protein arrays. *Anal Chem* **75:** (6) 1436.
Lin D, Tabb DL, Yates JR. 2003. Large-scale protein identification using mass spectrometry. *Biochim Biophys Acta* **1646:** (1-2) 1.
Liu CH, Ma WL, Shi R, Zhang B, Ou YQ, Zheng WL. 2003. Gene expression study of *Saccharomyces cerevisiae* with the Agilent 2100 Bioanalyser. *Br J Biomed Sci* **60:** (1) 22.
Lopez F, Pichereaux C, Burlet-Schiltz O, Pradayrol L, Monsarrat B, Esteve JP. 2003. Improved sensitivity of biomolecular interaction analysis mass spectrometry for the identification of interacting molecules. *Proteomics* **3:** (4) 402.
Luche S, Santoni V, Rabilloud T. 2003. Evaluation of nonionic and zwitterionic detergents as membrane protein solubilizers in two-dimensional electrophoresis. *Proteomics* **3:** (3) 249.
Michiels A, Van den Ende W, Tucker M, Van Riet L, Van Laere A. 2003. Extraction of high-quality genomic DNA from latex-containing plants. *Anal Biochem* **315:** (1) 85.
Miinea CP, Lienhard GE. 2003. Stoichiometry of site-specific protein phosphorylation estimated with phosphopeptide-specific antibodies. *Biotechniques* **34:** (4) 828.
Miller RD, Duan S, Lovins EG, Kloss EF, Kwok PY. 2003. Efficient high-throughput resequencing of genomic DNA. *Genome Res* **13:** (4) 717.
Pan SQ, Gu S, Bradbury EM, Chen X. 2003. Single peptide-based protein identification in human proteome through MALDI-TOF MS coupled with amino acids coded mass tagging. *Anal Chem* **75:** (6) 1316.
Pieper R, Su Q, Gatlin CL, Huang ST, Anderson NL, Steiner S. 2003. Multi-component immunoaffinity subtraction chromatography: An innovative step towards a comprehensive survey of the human plasma proteome. *Proteomics* **3:** (4) 422.
Rappsilber J, Moniatte M, Nielsen ML, Podtelejnikov AV, Mann M. 2003. Experiences and perspectives of MALDI MS and MS/MS in proteomic research. *Int J Mass Spectrom* **226:** (1) 223.
Rothemund DL, Locke VL, Liew A, Thomas TM, Wasinger V, Rylatt DB. 2003. Depletion of the highly abundant protein albumin from human plasma using the Gradiflow. *Proteomics* **3:** (3) 279.
Sunyaev S, Liska AJ, Golod A, Shevchenko A, Shevchenko A. 2003. MultiTag: Multiple error-tolerant sequence tag search for the sequence-similarity identification of proteins by mass spectrometry. *Anal Chem* **75:** (6) 1307.
T'Hoen PAC, De Kort F, Van Ommen GJB, Den Dunnen JT. 2003. Fluorescent labelling of cRNA for microarray applications: Article no. e20. *Nucleic Acids Res* **31:** (5) e20.
Thevis M, Loo RRO, Loo JA. 2003. In-gel derivatization of proteins for cysteine-specific cleavages and their analysis by mass spectrometry. *J Proteome Res* **2:** (2) 163.
Veenstra T, Conrads TP, Issaq HJ. 2003. An accurate mass tag strategy for quantitative and high throughput proteome measurements (Letter). *Instrum Sci Technol* **31:** (1) 103.
Wang YY, Cheng P, Chan DW. 2003. A simple affinity spin tube filter method for removing high-abundant common proteins or enriching low-abundant biomarkers for serum proteomic analysis. *Proteomics* **3:** (3) 243.
Xiong SX, Ding QX, Zhao ZW, Chen WZ, Wang GH, Liu SJ. 2003. A new method to improve sensitivity and resolution in matrix-assisted laser desorption/ionization time of flight mass spectrometry. *Proteomics* **3:** (3) 265.

### I6 Bioinformatics

Abe T, Kanaya S, Kinouchi M, Ichiba Y, Kozuki T, Ikemura T. 2003. Informatics for unveiling hidden genome signatures. *Genome Res* **13:** (4) 693.
Angulo J, Serra J. 2003. Automatic analysis of DNA microarray images

Copyright © 2003 John Wiley & Sons, Ltd. 

using mathematical morphology. *Bioinformatics* **19:** (5) 553.
Antoniadis A, Lambert-Lacroix S, Leblanc F. 2003. Effective dimension reduction methods for tumor classification using gene expression data. *Bioinformatics* **19:** (5) 563.
Bennett SP, Nevill-Manning CG, Brutlag DL. 2003. 3MOTIF: Visualizing conserved protein sequence motifs in the protein structure database. *Bioinformatics* **19:** (4) 541.
Berriz GF, White JV, King OD, Roth FP. 2003. GoFish finds genes with combinations of Gene Ontology attributes. *Bioinformatics* **19:** (6) 788.
Bicciato S, Luchini A, Di Bello C. 2003. PCA disjoint models for multiclass cancer analysis using gene expression data. *Bioinformatics* **19:** (5) 571.
Blekas K, Fotiadis DI, Likas A. 2003. Greedy mixture learning for multiple motif discovery in biological sequences. *Bioinformatics* **19:** (5) 607.
Brundno M, Do CB, Cooper GM, Kim MF, Davydov E, Green ED, Sidow A, Batzoglou S. 2003. LAGAN and Multi-LAGAN: Efficient tools for large-scale multiple alignment of genomic DNA. *Genome Res* **13:** (4) 721.
Camon E, Magrane M, Barrell D, Binns D, Fleischmann W, Kersey P, Mulder N, Oinn T, Maslen J, Cox A, Apweiler R. 2003. The gene ontology annotation (GOA) project: Implementation of GO in SWISS-PROT, TrEMBL, and InterPro. *Genome Res* **13:** (4) 662.
Chou KC, Elrod DW. 2003. Prediction of enzyme family classes. *J Proteome Res* **2:** (2) 183.
Datta S, Datta S. 2003. Comparisons and validation of statistical clustering techniques for microarray gene expression data. *Bioinformatics* **19:** (4) 459.
De la Nava JG, Santaella DF, Alba JC, Carazo JM, Trelles O, Pascual-Montano A. 2003. Engene: The processing and exploratory analysis of gene expression data. *Bioinformatics* **19:** (5) 657.
Giardine B, Elnitski L, Riemer C, Makalowska L, Schwartz S, Miller W, Hardison RC. 2003. GALA, a database for genomic sequence alignments and annotations. *Genome Res* **13:** (4) 732.
Guo FB, Ou HY, Zhang CT. 2003. ZCURVE: A new system for recognizing protein-coding genes in bacterial and archaeal genomes. *Nucleic Acids Res* **31:** (6) 1780.
Herrero J, Diaz-Uriarte R, Dopazo J. 2003. Gene expression data preprocessing. *Bioinformatics* **19:** (5) 655.
Holme P, Huss M, Jeong HW. 2003. Subnetwork hierarchies of biochemical pathways. *Bioinformatics* **19:** (4) 532.
Horner DS, Pesole G. 2003. The estimation of relative site variability among aligned homologous protein sequences. *Bioinformatics* **19:** (5) 600.
Iliopoulos I, Tsoka S, Andrade MA, Enright AJ, Carroll M, Poullet P, Promponas V, Liakopoulos T, Palaios G, Pasquier C *et al*. 2003. Evaluation of annotation strategies using an entire genome sequence. *Bioinformatics* **19:** (6) 717.
Jensen LJ, Gupta R, Staerfeldt HH, Brunak S. 2003. Prediction of human protein function according to Gene Ontology categories. *Bioinformatics* **19:** (5) 635.
Jewett AI, Huang CC, Ferrin TE. 2003. MINRMS: An efficient algorithm for determining protein structure similarity using root-mean-squared-distance. *Bioinformatics* **19:** (5) 625.
Johansson D, Lindgren P, Berglund A. 2003. A multivariate approach applied to microarray data for identification of genes with cell cycle-coupled transcription. *Bioinformatics* **19:** (4) 467.
Kano M, Nishimura K, Ishikawa S, Tsutsumi S, Hirota K, Hirose M, Aburatani H. 2003. Expression imbalance map: A new visualization method for detection of mRNA expression imbalance regions. *Physiol Genomics* **13:** (1) 31.
Kasturi J, Acharya R, Ramanathan M. 2003. An information theoretic approach for analyzing temporal patterns of gene expression. *Bioinformatics* **19:** (4) 449.
Kikuchi S, Tominaga D, Arita M, Takahashi K, Tomita M. 2003. Dynamic modeling of genetic networks using genetic algorithm and S-system. *Bioinformatics* **19:** (5) 643.
Le Blanc M, Kooperberg C, Grogan TM, Miller TP. 2003. Directed indices for exploring gene expression data. *Bioinformatics* **19:** (6) 686.
Lee K, Kohane IS, Butte AJ. 2003. PGAGENE: Integrating quantitative gene-specific results from the NHLBI Programs for Genomic Applications. *Bioinformatics* **19:** (6) 778.
Lee SA, Wormsley S, Kamoun S, Lee AFS, Joiner K, Wong B. 2003. An analysis of the *Candida albicans* genome database for soluble secreted proteins using computer-based prediction algorithms. *Yeast* **20:** (7) 595.
Luan YH, Li HZ. 2003. Clustering of time-course gene expression data using a mixed-effects model with B-splines. *Bioinformatics* **19:** (4) 474.
Lyons-Weiler J, Patel S, Bhattacharya S. 2003. A classification-based machine learning approach for the analysis of genome-wide expression data. *Genome Res* **13:** (3) 503.
Masseroli M, Cerveri P, Pelicci PG, Alcalay M. 2003. GAAS: Gene Array Analyzer Software for management, analysis and visualization of gene expression data. *Bioinformatics* **19:** (6) 774.
Matthiesen R, Lundsgaard M, Welinder KG, Bauw G. 2003. Interpreting peptide mass spectra by VEMS. *Bioinformatics* **19:** (6) 792.
Meyer F, Goesmann A, McHardy AC, Bartels D, Bekel T, Clausen J, Kalinowski J, Linke B, Rupp O, Giegerich R, Puhler A. 2003. GenDB: An open source genome annotation system for prokaryote genomes. *Nucleic Acids Res* **31:** (8) 2187.
Park T, Yi SG, Lee S, Lee SY, Yoo DH, Ahn JI, Lee YS. 2003. Statistical tests for identifying differentially expressed genes in time-course microarray experiments. *Bioinformatics* **19:** (6) 694.
Pertea G, Huang XQ, Liang F, Antonescu V, Sultana R, Karamycheva S, Lee Y, White J, Cheung F, Parvizi B, Tsai J, Quackenbush J. 2003. TIGR Gene Indices clustering tools (TGICL): A software system for fast clustering of large EST datasets. *Bioinformatics* **19:** (5) 651.
Presting GG. 2003. Mapping multiple co-sequenced T-DNA integration sites within the *Arabidopsis* genome. *Bioinformatics* **19:** (5) 579.
Rombauts S, Van de Peer Y, Rouze P. 2003. AFLPinSilico, simulating AFLP fingerprints. *Bioinformatics* **19:** (6) 776.
Sadowski MI, Parish JH. 2003. Automated generation and refinement of protein signatures: Case study with G-protein coupled receptors. *Bioinformatics* **19:** (6) 727.
Saito R, Suzuki H, Hayashizaki Y. 2003. Construction of reliable protein-protein interaction networks with a new interaction generality measure. *Bioinformatics* **19:** (6) 756.
Shih ESC, Hwang MJ. 2003. Protein structure comparison by probability-based matching of secondary structure elements. *Bioinformatics* **19:** (6) 735.
Shmulevich I, Astola J, Cogdell D, Hamilton SR, Zhang W. 2003. Data extraction from composite oligonucleotide microarrays - Online art no. e36. *Nucleic Acids Res* **31:** (7) e36.
Stern MD, Anisimov SV, Boheler KR. 2003. Can transcriptome size be estimated from SAGE catalogs? *Bioinformatics* **19:** (4) 443.
Sturn A, Mlecnik B, Pieler R, Rainer J, Truskaller T, Trajanoski Z. 2003. Client-Server environment for high-performance gene expression data analysis. *Bioinformatics* **19:** (6) 772.
Subramanian S, Madgula VM, George R, Mishra RK, Pandit MW, Kumar CS, Singh L. 2003. Triplet repeats in human genome: Distribution and their association with genes and other genomic regions. *Bioinformatics* **19:** (5) 549.
Subramanian S, Mishra RK, Singh L. 2003. Genome-wide analysis of Bkm sequences (GATA repeats): Predominant association with sex chromosomes and potential role in higher order chromatin organization and function. *Bioinformatics* **19:** (6) 681.
Tillier ERM, Lui TWH. 2003. Using multiple interdependency to separate functional from phylogenetic correlations in protein alignments. *Bioinformatics* **19:** (6) 750.
Toppo S, Cannata N, Fontana P, Romualdi C, Laveder P, Bertocco E, Lanfranchi G, Valle G. 2003. TRAIT (TRAnscript Integrated Table): a knowledgebase of human skeletal muscle transcripts. *Bioinformatics* **19:** (5) 661.
Trost E, Hackl H, Maurer M, Trajanoski Z. 2003. Java editor for biological pathways. *Bioinformatics* **19:** (6) 786.
Venet D. 2003. MatArray: A Matiab toolbox for microarray data. *Bioinformatics* **19:** (5) 659.
Wang JX, Liang P. 2003. DigiNorthern, digital expression analysis of query genes based on ESTs. *Bioinformatics* **19:** (5) 653.
Wong P, Kolesov G, Frishman D, Houry WA. 2003. Phylogenetic Web Profiler. *Bioinformatics* **19:** (6) 782.
Wu YM, Wang XY, Liu X, Wang YF. 2003. Data-mining approaches reveal hidden families of proteases in the genome of malaria parasite. *Genome Res* **13:** (4) 601.
Ye YZ, Jaroszewski L, Li WZ, Godzik A. 2003. A segment alignment approach to protein comparison. *Bioinformatics* **19:** (6) 742.
Zhang CT, Zhang R, Ou HY. 2003. The Z curve database: A graphic representation of genome sequences. *Bioinformatics* **19:** (5) 593.

Copyright © 2003 John Wiley & Sons, Ltd. 
